# Valgus osteotomy by external fixation for treatment for developmental coxa vara

**DOI:** 10.1007/s11751-013-0178-3

**Published:** 2013-10-01

**Authors:** Hany Hefny, Elhussein Mohamed Elmoatasem, Wael Nassar

**Affiliations:** Ain Shams University, Cairo, Egypt

**Keywords:** Coxa vara, Osteotomy, External fixator, Ilizarov

## Abstract

Valgus subtrochanteric osteotomy is the standard surgical treatment for coxa vara. Nevertheless, there is no consensus on the method of fixation and osteotomy technique. There are some reports on employing rigid internal fixation methods that preclude the need of postoperative immobilization. This is a technical description of a valgus osteotomy performed using external fixation with preoperative and postoperative data on a cohort of 9 patients. In this study, 9 hips in 9 patients with the diagnosis of developmental coxa vara underwent a subtrochanteric osteotomy with stabilization by an external fixator. The planned correction angle was obtained for all 9 patients with the osteotomies healing primarily. Radiographic analysis showed an improvement in Hilgenreiner’s epiphyseal angle and the neck-shaft angle. There were no major complications associated with use of this method of stabilization. Minimal access surgery using external fixation for a valgus osteotomy of the proximal femur is safe and effective for the treatment for coxa vara and limb length discrepancy. It has potential advantages over commonly used open techniques and provides available alternative to currently applied methods used for fixation of proximal femoral osteotomies.

## Introduction

Classically defined as a femoral neck-shaft angle of <110°, coxa vara is relatively uncommon and is present in approximately 1/25,000 children [1]. This deformity results from a heterogeneous group of conditions that can be classified as congenital, developmental, dysplastic and traumatic [1]. The natural history of coxa vara may be debilitating as the child develops progressive limb length discrepancy, limp, pain, abductor weakness, and restricted motion. Secondary acetabular dysplasia and genu valgum may compound the problem. With the exception of some forms of developmental coxa vara which can resolve, a variety of surgical methods have evolved to deal with progressive coxa vara [1–5]. Despite well-executed osteotomies, recurrence is cited in the literature as ranging from 30 to 70 % [1, 3, 4, 6]. The high recurrence rate can be explained by the biomechanics of the underlying disorder. Coxa vara lends itself to progression as the physis assumes a more vertical position. Resultant forces across the hip then become shearing rather than compressive [7]. This bending moment is damaging not only to the mechanical properties of stability of the epiphysis but also to normal continued physeal growth. Thus, unlike the normal hip where these resultant forces are compressive, in coxa vara, the shearing forces cause the deformity to recur unless the osteotomy addresses the physeal position satisfactorily [8]. Adequate surgical correction of coxa vara can be difficult, requiring careful clinical and radiographic assessment, preoperative planning, proper implant selection and meticulous surgical technique. Restoration of the femoral capital physis to a relatively horizontal position will aid to normalize the biomechanical forces. This correction of Hilgenreiner’s epiphyseal angle (HEA) to <38° is the goal of intraoperative correction. This has been shown to reduce the risk of recurrent coxa vara, regardless of the etiology of the deformity and the age of the patient [4, 8]. Achieving corrections of limb deformities and length discrepancies through less invasive means is increasingly popular [9]. Recently, good results have been reported using external fixator systems for the correction of proximal femoral deformities secondary to slipped capital femoral epiphysis (SCFE), Perthes’ disease in children and percutaneous proximal femoral osteotomy for coxa vara [10–12]. We describe the surgical technique of a minimally invasive percutaneous approach for correction of severe coxa vara using external fixation.

## Patients and methods

**Fig. 1:**
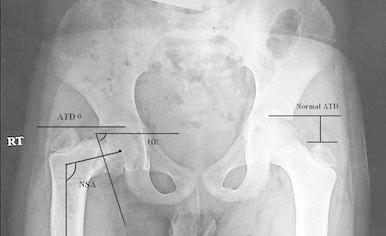
Radiographic evaluations on the standing anteroposterior radiographs with the hips in neutral position were performed. *a* Measurements of the Hilgenreiner’s epiphyseal angle (HE), neck-shaft angle (NSA), articulo-trochanteric distance (ATD)

Between 2002 and 2010, nine subtrochanteric femoral osteotomies were performed in nine consecutive patients for treatment for coxa vara using external fixator systems for stabilization. Two different types of external fixation were used: the monolateral limb reconstruction system (LRS) fixator in 2 cases and the multiplanar Ilizarov fixator in 7 cases. The age at initial surgery was averaged 10.1 years (range 6–16 years). All the patients included in the study had developmental coxa vara. Any patient with coxa vara due to other etiologies, viz, acquired, dysplastic or congenital (e.g., fracture neck femur, fibrous dysplasia or proximal femoral focal deficiency, respectively) was excluded from the study. Patients included in this study presented with the chief complaint of a limp, with minimal or no pain. Physical examination revealed a short leg gait with an abductor lurch, a positive Trendelenburg test, limitation of abduction and internal rotation of the involved hip in all patients. Standard radiographs, including an anteroposterior (AP) view of the pelvis and frog lateral of the affected hip, were done before and 1, 3, 6 and 12 months after surgery. Limb scanograms were available preoperatively in all patients. HEA and the neck-shaft angle were measured before surgery, immediately after surgery and at latest follow-up on the AP pelvis radiograph. All patients had a HEA of more than 60°, a femoral neck-shaft angle (FNSA) of <95° and an obliterated or reversed articulo-trochanteric distance (ATD, Fig. 1). Six patients had preoperative shortening averaging 3.4 cm (range 2–5). In 3 patients, an additional diaphyseal femoral osteotomy was done to correct limb length discrepancy and mechanical axis deviation (Fig. 1). The mean duration of follow-up was 4.2 years (range 2–8 years).

### Operative technique

**Fig. 2:**
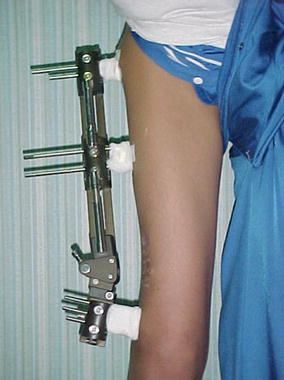
Clinical photograph showing application of Orthofix fixator after distal femoral osteotomy for limb lengthening and correction of mechanical axis

**Fig. 3:**
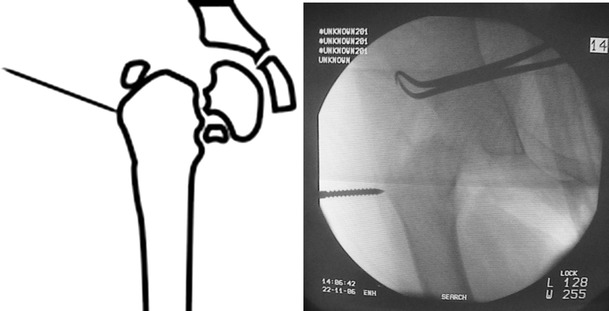
Application of proximal half-pin in direction from superolateral to inferomedial

**Fig. 4:**
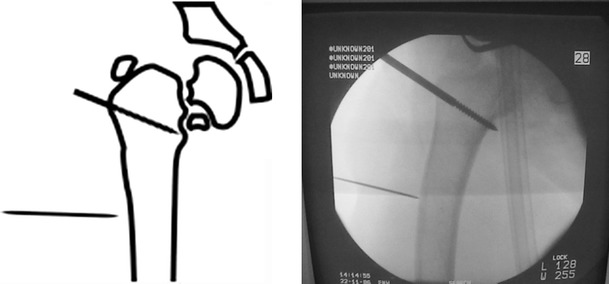
Distal half-pin inserted in a perpendicular angle with the anatomical axis of the proximal femur

All patients were operated under general anesthesia. Patients were positioned in lateral decubitus. The involved lower extremity was prepared and draped. With the affected limb held in hip neutral position, a true AP view of the involved hip was reproduced on the C-arm monitor. Placing the half-pins in the proximal segment with the limb in the hip neutral position avoids the need for extensive skin release around the half-pins after the corrective osteotomy. The proximal half-pin was placed in a direction from superolateral to inferomedial. We used a modified technique by inserting a Kirschner wire, followed by cannulated drill and finally the half-pin inserted under C-arm control (Fig. 2). In the case of using the Ilizarov fixator, the half-pins were mounted on femoral arch and care was taken to allow at least 2 finger breadths between the arch and the underlying skin (Fig. 3). Next, with the limb in neutral alignment in the frontal, sagittal and transverse planes (i.e., knee neutral position), distal half-pins to the osteotomy site were placed at right angles to the femoral shaft, mounted on one or more arches according to the size of the limb (Fig. 4). For better control of the proximal fragment of the femur and to avoid uncontrolled displacement, the frame was mounted and the connecting rods between the 2 arches were not tightened until the osteotomy was made.

**Fig. 5:**
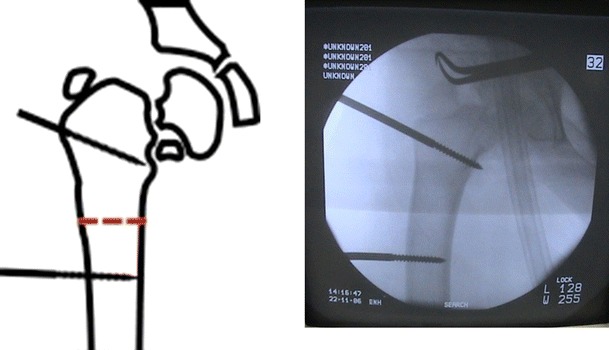
Osteotomy site at the subtrochanteric area

**Fig. 6:**
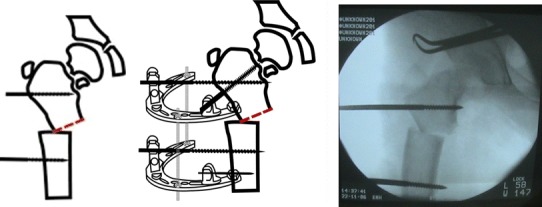
Acute correction through a subtrochanteric osteotomy

**Fig. 7:**
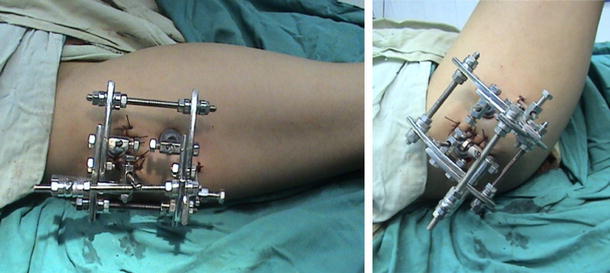
Clinical photograph showing the application of 2 arches and 3 connecting rods

A 2-cm transverse incision was made at the level of the proposed osteotomy site in the subtrochanteric area. Multiple drill holes were made, which were then connected by an osteotome or using a Gigli saw (Fig. 5). Once the osteotomy was complete, the correction was achieved by approximating the 2 arches using the 3 connecting rods so that the arches became parallel or by using the swiveling clamp on the LRS fixator (Figs. 6, 7).

## Results

**Fig. 8:**
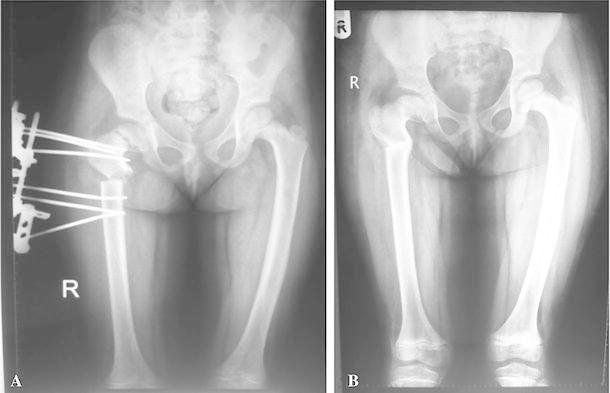
**a** X-ray for postoperative follow-up of osteotomy after acute correction, **b** healed osteotomy with correction of neck-shaft angle

A total of 9 subtrochanteric osteotomies were performed with coxa vara of the same etiology. One patient had revision surgery for a failed subtrochanteric osteotomy with plate and screws. All osteotomies achieved the planned correction angle (Fig. 8). Radiographic analysis revealed an average correction of Hilgenreiner’s epiphyseal angle by 41.3° (range 30°–64°) from 75.2° (range 60°–102°) before surgery to 33.8° (range 30°–38° degrees) after surgery. The FNSA improved by an average of 48.2° (range 45°–55°); this was from an average of 82° (range 70°–95°) before surgery to an average of 132.3° (range 125°–140°) after surgery. The ATD improved from −8 mm (range −12 to 1 mm) before surgery to +10 mm (range +8 to +18 mm) after surgery. The minimum follow-up was 2 years. At latest follow-up, no loss of correction was measured.

There were no intraoperative fractures or neurovascular injuries. Evaluation of follow-up radiographs showed that all osteotomies had healed by 4 months after surgery with no nonunions, malunions, device failures or avascular necrosis. Position changes in bony fragments were not noted in any patients after surgery. Complications occurred in 4 (44.4 %) of 9 hips. Three (33.3 %) hips had postoperative pin tract infections; 2 (22.2 %) were superficial; and 1 (11.1 %) was deep. All five superficial infections were treated by intravenous administration of antibiotics and frequent dressing changes and healed uneventfully. In the deep infection case, removal of the half-pin and debridement were performed 2 months after the primary operation, which did not jeopardize the fixation of the frame.

## Discussion

Multiple surgical techniques have been described for correction of coxa vara. These include the Langenskiold intertrochanteric osteotomy, the interlocking intertrochanteric osteotomy, the valgus subtrochanteric osteotomy with blade plate fixation and the Pauwel’s *Y-*shaped intertrochanteric osteotomy [3, 4, 6, 8, 13–15]. Excellent long-term follow-up has been reported with both the Pauwel’s osteotomy and valgus subtrochanteric osteotomy fixed with a blade plate [4, 6, 8]. Desai and Johnson reported excellent long-term results of treatment for congenital coxa vara utilizing a valgus subtrochanteric osteotomy in 20 hips of 12 patients [8]. Their mean postoperative correction of the FNSA to 136° and the HEA to 30° is comparable with our series (132.3° and 33.8°, respectively). Outstanding long-term results of the Pauwel’s osteotomy were reported by Cordes et al. in a series of 14 children and 18 hips with coxa vara of multiple etiologies [6]. Their mean postoperative correction of the FNSA to 141° and the HEA to 29° was comparable to the results of this series. Recurrence of deformity occurred in a single case in that series due to loss of fixation postoperatively; this did not occur in our group of patients.

There are several pitfalls with current techniques for proximal femoral osteotomies. These include the need for an open procedure with removal of a trapezoidal fragment of bone from the subtrochanteric area, producing blood loss and further shortening of an already short extremity [15]. There are limited choices of implants to allow secure fixation of the underlying bone, which can be quite small in young children. Furthermore, any fixation device needs to avoid the proximal femoral growth plate, leaving a limited length of bone available for secure fixation. Typically, the implant is rigidly applied to the underlying bone, making appropriate lateral translation of the distal fragment and minor adjustments after fixation very difficult. Depending on the stability achieved at surgery, some of these children may need a hip spica cast for several weeks after surgery to protect against displacement at the osteotomy site. All will need a second operation to remove the internal fixation device [3].

The ideal fixation device for a multiplanar femoral trochanteric osteotomy is one that allows the surgeon to perform an accurate correction, is easily applied, maintains rigid fixation, permits early joint motion and mobilization of the patient and avoids another operation for removal [10]. The external fixator technique fits this description. There are several potential benefits of this technique, which include avoidance of a large open exposure, decreased potential for significant blood loss and the ability to achieve an accurate and sustained correction of the multiplanar deformity. Using a low-energy osteotomy in this technique, limb length discrepancy can be improved without compromising the quality and time for bony union. Early mobilization with a short hospital stay is possible by avoiding the need for any supplemental cast immobilization. Problems associated with internal fixation such as prominent hardware, implant failure, the possibility of violating the proximal femoral growth plate, the need for a second major surgical procedure for removing an internal implant and the potential for deep infection are significantly decreased. However, there are potential obstacles to this technique. These include a need to be familiar with the use of external fixators capable of using deformity correction, e.g., the Ilizarov fixator or Orthofix LRS fixator, although other external fixator systems can be used as long as the principles outlined above are followed. The inconvenience of pin sites with the possibility of drainage or infection around the pins is another drawback and must be discussed with the patient and relatives beforehand. By using hydroxyapatite-coated half-pins, our modified technique of pin insertion [16], avoiding thermal necrosis while drilling, oral antibiotics early for pin site drainage and doing appropriate pin site releases and care, we have recorded few deep pin-related complaints. With preoperative education and counseling, the patients adapt well to the external fixator.

Despite well-performed osteotomies, the literature cites recurrence rates of 30–70 % [9, 16]. This is in contrast to this report where none of the 9 hips had to be revised. In a study of valgus osteotomies for coxa vara, Carroll et al. reported a recurrence rate of nearly 50 % [6]. If the Hilgenreiner’s epiphyseal angle is corrected to 38° or less, 95 % of the children had no recurrence of their varus deformity [6]. The most important factor in reducing the likelihood of recurrent varus is restoration of the femoral neck physis to an anatomic position (a HE angle of 38° or less), thereby normalizing the forces across the physis [4, 8]. It may be to achieve an overcorrection of the HE angle to the normal (anatomic) value of 22° to ensure no recurrences. Although we report no recurrences until the latest follow-up, a weakness in this study is a longer minimum follow-up period for all patients in order to fully assess the long-term impact of our technique on the incidence of recurrence.

In the event a repeat osteotomy is required, this technique avoids the increased morbidity from the absence of large incisions or retained hardware. This technique may also have a role in the treatment for other proximal femoral deformities in children such as those associated with SCFE, Perthes’ disease and developmental dysplasia of the hip.

The valgus osteotomy produced a reduction in leg length discrepancy, but 3 patients required a distal femoral osteotomy to address additional length discrepancies and angular deformities near the knee. Shim et al. [17] noted that patients with progressive coxa vara often develop ipsilateral compensatory genu valgum. This highlights the need to avoid medial displacement of the osteotomy, which will exacerbate loading of the lateral compartment and distal femoral physis. This problem has not been addressed in more recent articles on the subject, such as those by Sabharwal et al. [12], Skaggs et al. [18] and Kim et al. [19]. When the coxa vara is corrected, the genu valgum may be unmasked and therefore recommend a full-length standing radiograph or CT scanogram to document alignment and leg length problems preoperatively. In this study, we addressed mechanical axis correction by subtrochanteric and distal femoral osteotomies enabling correction of the coxa vara, mechanical axis deviation and limb length inequality.

## Conclusion

A percutaneous external fixator-based technique is described for the treatment for developmental coxa vara and limb length discrepancy in a pediatric cohort. It has potential advantages over commonly used open techniques in being minimally invasive, easily reproducible and provides a versatile alternative to currently available methods for fixation of proximal femoral osteotomies.
